# The Burden of Urticaria in the Middle East and North Africa Region From 1990 to 2021: A Systematic Analysis Based on the GBD Study 2021

**DOI:** 10.1002/hsr2.72976

**Published:** 2026-08-04

**Authors:** Amir Sajad Bagheryan, Soroush Oraee, Mehra Fekri, Farzin Tahmasbi Arashlow, Hojjat Layegh, Seyed Aria Nejadghaderi, Shirin Zaresharifi

**Affiliations:** ^1^ Student Research Committee, School of Medicine Shahid Beheshti University of Medical Sciences Tehran Iran; ^2^ Medical Students Research Centre Iran University of Medical Sciences Tehran Iran; ^3^ Department of Plastic Surgery Shahid Beheshti University of Medical Sciences Tehran Iran; ^4^ HIV/STI Surveillance Research Center, and WHO Collaborating Center for HIV Surveillance, Institute for Futures Studies in Health Kerman University of Medical Sciences Kerman Iran; ^5^ Skin Research Center Shahid Beheshti University of Medical Sciences Tehran Iran

**Keywords:** burden of disease, disability­ adjusted life years, incidence, Middle East and North Africa, prevalence, urticaria

## Abstract

**Background and Aims:**

Urticaria is a condition which can impair the quality of life of affected individuals. There is a lack of recent studies on the burden of this disease in the Middle East and North Africa (MENA) region. This article reported the incidence, prevalence, and disability‐adjusted life years (DALYs) of urticaria from 1990 to 2021 in the MENA region, by age, sex, and socio‐demographic index (SDI).

**Methods:**

We retrieved data from the Global Burden of Disease (GBD) study 2021 on the incidence, prevalence, and DALYs due to urticaria in the MENA region. They were estimated using the DisMod‐MR 2.1 tool. The values were presented as total counts and age‐standardized rates per 100,000 individuals alongside their 95% uncertainty intervals (UIs).

**Results:**

In 2021, the age‐standardized incidence rate was 1649.2 (95% UI: 1452.7–1854.3), the point prevalence was 936.1 (95% UI: 825.8–1058.6) and the DALY rate was 56.1 (95% UI: 36.8–79.2) per 100,000 in MENA region, with no significant changes since 1990. Egypt displayed the highest burden of urticaria, while Turkey had the lowest one. The incident cases and rates were highest in younger ages with the peak observed in children under‐5 years old with higher incidence in women across all ages. Countries with mid‐level SDI had the highest age‐standardized DALY rates.

**Conclusion:**

Overall, the burden of urticaria has not changed significantly since 1990. Studies on standardizing reporting to strengthen future burden estimates, raising awareness and improving access to healthcare resources and lastly reducing exposure to environmental factors such as pollutants and allergens are recommended.

## 1 Introduction

Urticaria is a common inflammatory skin disease characterized by transient, pruritic, erythematous swelling caused by mast cell degranulation and histamine release. These reactions can occur in the superficial dermis, causing wheals, or extend into the deep dermis and submucosa, leading to angioedema. The disease may present as wheals, angioedema, or a combination of both [1]. The condition affects up to 20% of the global population at some point in their lives, with chronic urticaria impacting approximately 1% of individuals worldwide [2, 3].

The clinical manifestations of urticaria, including intense itching and discomfort, can significantly impair patients’ quality of life, leading to sleep disturbances, anxiety, and reduced productivity [4, 5]. Notably, the burden of urticaria is not uniformly distributed across different regions and populations. Studies have shown that females and children aged 1–4 years are more commonly affected than males and adults, respectively [6]. Additionally, regions with lower gross domestic product per capita tend to experience higher prevalence and incidence rates of urticaria, suggesting socioeconomic factors may influence disease burden [6].

In 2019, a total of 65 million individuals were affected by urticaria with a disability‐adjusted life years (DALYs) rate of 50.4 per 100,000 people [7]. Despite its high prevalence and associated morbidity, comprehensive data on the burden of urticaria, particularly in specific regions remain limited. Previous studies mostly focus on the global and regional burden of urticaria [7, 8, 9], but no study with a focus on the Middle East and North Africa (MENA) region has been recently published. The MENA region is a heterogeneous and highly developing part of the world, including countries with some of the highest and lowest human development index globally [10]. Ongoing conflicts, climate change, and high economic growth rates in some countries make it necessary to have updated and comprehensive data on the burden of disease across different countries in this region [11, 12]. The Global Burden of Disease (GBD) Study provides a systematic analysis of available data on various diseases and injuries worldwide and offers burden of these through different epidemiological metrics [13]. Understanding the epidemiology and burden of urticaria in the MENA region is crucial for several reasons. It helps to identify high‐risk populations and design public health interventions accordingly. Moreover, by analyzing the differences in epidemiological trends, governments and policymakers may be able to develop policies to control them. Finally, understanding the burden of urticaria would help policymakers determine how much resources should be allocated to addressing this disease [14]. This study aimed to assess the burden of urticaria in the MENA region from 1990 to 2021, using data from the GBD 2021. We reported the incidence, prevalence, and DALYs over this period in the region. Additionally, we reported DALYs in relation to the socio‐demographic index (SDI).

## 2 Methods

### 2.1 Overview

This study adheres to the Guidelines for Accurate and Transparent Health Estimates Reporting (GATHER) [15] and the Strengthening the Reporting of Observational Studies in Epidemiology (STROBE) guidelines [16]. We used data from the GBD 2021 study provided by the Institute for Health Metrics and Evaluation. This data estimates the burden of 371 diseases and injuries, including urticaria, for 204 countries and territories, through metrics such as prevalence, incidence, DALYs, and deaths by age groups, sex, location, and year from 1990 to 2021 [17]. This study focused on the burden of urticaria, assessing national and regional rates and trends over the specified period.

### 2.2 Data Sources

The data for the MENA region and its 21 countries of Afghanistan, Algeria, Bahrain, Egypt, Iraq, Iran, Jordan, Kuwait, Lebanon, Libya, Morocco, Oman, Palestine, Qatar, Saudi Arabia, Sudan, Syrian Arab Republic, Tunisia, Turkey, United Arab Emirates (UAE), and Yemen are sourced from the GBD 2021 results tool, available at https://vizhub.healthdata.org/gbd-results/. This information is derived from various sources [13]. In GBD 2021, relevant data were gathered through a systematic review of the literature to identify epidemiological data on urticaria. Studies were included if they met the following criteria: they were published between 1980 and 2012, reported data on the incidence or prevalence of urticaria, utilized samples representative of the general population (excluding those derived from clinical trials or dermatology clinic‐based studies), had a sample size exceeding 100 participants, and provided sufficient methodological details and sample characteristics to allow for quality assessment. For GBD 2016, the search strategy from GBD 2010 was replicated to identify epidemiological studies published between 2013 and 2016. More detailed explanations of data sources are available in previous studies [13].

### 2.3 Case Definitions and Burden Estimates

GBD 2021 defines urticaria as a skin rash triggered by a reaction to food, medicine, or other irritants following the International Classification of Disease (ICD)−10 code of L50‐L50.9 and ICD‐9 codes of 708‐708.9 [13, 18].

The methods for estimating the burden of urticaria have been previously described [13]. Briefly, prevalence, incidence, and DALYs of urticaria were estimated using DisMod‐MR 2.1, a Bayesian meta‐regression tool for modeling disease epidemiology. The primary data sources consisted predominantly of prevalence estimates, with a limited number of incidence data points. For GBD 2017, both prevalence and incidence estimates were generated using a 25‐year time window. Mortality was set to zero as urticaria does not cause death, and remission rates were constrained to a range of 0.5–2, implying an estimated duration of 6–8 months. In GBD 2021, within‐DisMod crosswalks were replaced by crosswalks performed using the MR‐BRT modeling tool. Specific data points were excluded as outliers if they were identified as overestimates or underestimates when compared to country, regional, or global patterns. Disability weights (DWs) were used to calculate DALY of urticaria. DWs quantify the severity of health conditions on a scale from 0 (perfect health) to 1 (equivalent to death). For urticaria, which does not cause mortality, the burden is calculated only through years lived with disability (YLD). Mild urticaria had a DW of 0.027 (95% confidence interval [CI]: 0.015–0.042), reflecting slight visible physical deformity with occasional soreness or itching, causing minor worry and discomfort. Severe urticaria had a DW of 0.188 (95% CI: 0.124–0.267), indicating significant visible deformity, soreness, and itching, along with social stigma and difficulty sleeping and concentrating.

### 2.4 Statistical Analysis and Presentation of Estimates

All estimates are presented as total counts and age‐standardized rates per 100,000 individuals. Age standardization involves applying age‐specific rates from a study population to the GBD standard population. Changes in estimates from 1990 are reported as percentages. All metrics are reported alongside a 95% uncertainty interval (UI), defined as the 2.5th and 97.5th percentile of 500 draws for each estimate [13]. Estimates are further reported by corresponding sex and age groups. The association between age‐standardized DALY rates and SDI was investigated using smoothing spline models [19]. SDI is a summary measure ranging from zero to one with higher values indicating a more developed socio‐demographic status and is strongly correlated with health outcomes. It is computed by obtaining the geometric mean of three normalized indicators: lag‐distributed income per capita, average years of educational attainment among people older than 15 years, and the total fertility rate among women younger than 25 [20]. An SDI value of zero indicates minimal development with poor income, low education, and high fertility, while a value of one represents maximum development with high income, advanced education, and low fertility. All visual representations were created with R software (Version 4.4.2). The reporting of statistical analyses in this study follows the recommendations of Assel et al. for transparent and appropriate statistical reporting in clinical research [21].

## 3 Results

### 3.1 The MENA Region

In 2021, the age‐standardized incidence rate (ASIR) was 1649.2 (95% UI: 1452.7–1854.3) per 100,000 population, reflecting no significant changes since 1990. The age‐standardized point prevalence of urticaria in 2021 was 936.1 (95% UI: 825.8–1058.6) per 100,000 population, showing no significant changes between 1990 and 2021. Likewise, the age‐standardized DALY rate was 56.1 (95% UI: 36.8–79.2) per 100,000, with no significant changes since 1990 (Table 1).

**Table 1:** Prevalent cases, incident cases, and DALYs, along with their age‐standardized rates, due to urticaria in 2021 for both sexes, and the percentage change in rates per 100,000 population from 1990 to 2021 in Middle East and North Africa.

	Incidence (95% UI)			Prevalence (95% UI)			DALYs (95% UI)		
Location	Counts (2021)	Rate (2021)	Pcs in rate 1990–2021	Counts (2021)	Rate (2021)	Pcs in rate 1990–2021	Counts (2021)	Rate (2021)	Pcs in rate 1990–2021
Middle East and North Africa	10,333,263.8 (9,078,983.9, 11,654,636.9)	1649.2 (1452.7, 1854.3)	0.1 (−0.2, 0.3)	5,879,513.1 (5,167,959.6, 6,683,121.6)	936.1 (825.8, 1058.6)	0.1 (−0.2, 0.4)	353,237.2 (231,209.4, 499,205.3)	56.1 (36.8, 79.2)	0.1 (−1.5, 1.8)
Afghanistan	576,762.0 (496,368.3, 672,726.2)	1605.0 (1417.9, 1808.9)	−1.0 (−1.3, −0.7)	323,726.0 (279,661.3, 379,362.7)	909.7 (799.0, 1038.0)	−1.0 (−1.3, −0.7)	19,589.9 (13,026.9, 27,933.8)	54.1 (35.9, 76.0)	−0.8 (−6.0, 4.7)
Algeria	720,142.2 (633,971.8, 814,639.7)	1602.3 (1415.6, 1805.7)	−0.2 (−0.2, −0.1)	408,611.6 (357,700.9, 467,345.8)	908.1 (797.5, 1036.6)	−0.2 (−0.2, −0.1)	24,551.3 (16,041.2, 34,723.7)	54.5 (35.7, 77.3)	−0.1 (−5.0, 5.0)
Bahrain	21,985.4 (19,425.1, 24,613.9)	1557.2 (1373.4, 1764.2)	−1.1 (−1.3, −0.9)	12,524.0 (10,967.9, 14,127.1)	882.1 (774.2, 1010.4)	−1.1 (−1.3, −0.9)	753.4 (492.1, 1071.2)	53.1 (35.1, 75.6)	−0.8 (−6.1, 4.4)
Egypt	2,111,979.2 (1,819,602.1, 2,411,970.6)	1892.4 (1642.7, 2130.7)	−0.2 (−0.2, −0.1)	1,209,278.5 (1,045,096.3, 1,385,477.8)	1084.3 (942.1, 1224.4)	−0.2 (−0.2, −0.1)	73,170.0 (47,364.9, 104,219.8)	65.2 (42.4, 92.6)	−0.1 (−5.1, 5.0)
Iran	1,380,916.3 (1,224,234.3, 1,558,420.7)	1693.5 (1500.1, 1929.4)	−0.1 (−0.1, −0.1)	788,149.8 (694,326.5, 890,103.4)	959.0 (845.8, 1089.8)	−0.1 (−0.1, −0.1)	47,084.0 (31,033.8, 66,111.2)	57.5 (38.0, 80.9)	0.0 (−5.4, 6.0)
Iraq	675,046.4 (589,972.5, 769,711.3)	1598.1 (1411.7, 1801.9)	0.0 (−0.1, 0.0)	383,727.5 (334,740.4, 442,130.7)	905.7 (795.3, 1034.4)	0.0 (−0.1, 0.0)	23,098.2 (15,213.4, 32,708.8)	54.2 (35.9, 76.3)	0.1 (−1.4, 1.5)
Jordan	194,991.0 (171,215.1, 222,347.9)	1589.7 (1403.8, 1794.2)	−0.4 (−0.5, −0.3)	110,978.5 (97,164.8, 127,013.2)	900.8 (790.9, 1029.6)	−0.4 (−0.5, −0.3)	6691.2 (4371.6, 9588.8)	54.1 (35.6, 76.4)	−0.4 (−5.9, 5.1)
Kuwait	68,408.1 (60,076.3, 76,661.4)	1592.7 (1406.8, 1795.6)	1.3 (1.0, 1.7)	38,999.1 (34,172.2, 43,883.3)	902.5 (792.3, 1030.9)	1.3 (1.0, 1.7)	2329.7 (1509.0, 3272.1)	54.2 (35.6, 77.3)	0.8 (−4.5, 7.0)
Lebanon	84,357.8 (74,751.2, 94,184.4)	1602.5 (1415.9, 1805.5)	−0.4 (−0.6, −0.2)	48,022.8 (42,263.6, 54,107.8)	908.2 (797.9, 1036.3)	−0.4 (−0.6, −0.2)	2860.1 (1898.2, 4078.2)	54.3 (35.9, 77.9)	−0.4 (−5.7, 4.7)
Libya	102,907.4 (91,166.3, 115,213.8)	1600.7 (1413.9, 1804.6)	0.3 (0.2, 0.5)	58,728.8 (51,674.7, 66,377.1)	907.2 (796.6, 1035.8)	0.3 (0.2, 0.6)	3507.0 (2334.4, 4985.6)	54.3 (36.0, 77.2)	0.0 (−5.3, 5.0)
Morocco	584,121.8 (517,143.4, 655,526.2)	1604.6 (1417.7, 1808.0)	−0.3 (−0.3, −0.2)	331,928.4 (292,292.7, 376,073.0)	909.4 (798.6, 1038.1)	−0.3 (−0.3, −0.3)	19,867.4 (13,013.2, 28,137.1)	54.5 (35.8, 77.2)	−0.4 (−5.8, 5.5)
Oman	72,533.8 (63,425.2, 81,960.7)	1565.0 (1380.6, 1772.9)	0.2 (0.1, 0.3)	41,225.2 (35,740.3, 47,022.4)	886.7 (778.6, 1015.6)	0.2 (0.1, 0.3)	2488.5 (1645.7, 3493.3)	53.3 (35.4, 74.3)	0.3 (−5.3, 6.0)
Palestine	86,887.3 (75,268.0, 99,645.7)	1603.0 (1416.2, 1806.5)	−0.4 (−0.6, −0.3)	49,306.8 (42,929.8, 57,153.1)	908.5 (797.8, 1037.3)	−0.4 (−0.6, −0.3)	2978.6 (1958.9, 4236.7)	54.4 (36.0, 75.7)	−0.6 (−6.2, 5.2)
Qatar	41,680.5 (36,114.8, 47,165.7)	1537.2 (1354.1, 1745.1)	0.2 (0.1, 0.3)	23,637.9 (20,517.0, 26,930.1)	870.7 (764.2, 999.4)	0.2 (0.1, 0.3)	1422.5 (926.6, 1989.5)	52.4 (34.7, 73.9)	0.0 (−5.1, 5.9)
Saudi Arabia	554,735.0 (488,491.9, 623,281.6)	1568.8 (1384.7, 1772.9)	−0.3 (−0.4, −0.2)	315,850.3 (276,298.1, 356,684.9)	888.8 (780.0, 1016.7)	−0.3 (−0.4, −0.2)	18,933.0 (12,482.2, 26,900.2)	53.3 (35.4, 76.4)	−0.1 (−5.1, 6.0)
Sudan	747,563.7 (645,397.9, 859,465.9)	1601.3 (1415.1, 1803.6)	−0.2 (−0.2, −0.1)	423,265.4 (368,025.0, 491,540.9)	907.5 (797.0, 1035.8)	−0.2 (−0.3, −0.1)	25,551.0 (16,796.6, 37,336.1)	54.3 (35.9, 77.8)	0.3 (−4.9, 6.0)
Syrian Arab Republic	216,456.3 (191,791.7, 245,385.5)	1621.5 (1433.4, 1822.8)	1.3 (1.0, 1.6)	123,430.8 (109,372.8, 140,647.1)	919.2 (807.3, 1047.3)	1.3 (1.0, 1.6)	7364.0 (4882.5, 10,433.8)	54.9 (36.4, 77.7)	0.9 (−4.1, 6.2)
Tunisia	181,043.8 (160,797.0, 201,782.7)	1604.5 (1417.9, 1806.9)	0.0 (0.0, 0.1)	103,119.3 (91,209.0, 116,231.2)	909.4 (798.8, 1037.4)	0.0 (0.0, 0.1)	6150.6 (4098.3, 8734.7)	54.5 (36.3, 77.6)	−0.2 (−5.6, 5.4)
Turkey	1,180,928.7 (1,049,639.3, 1,316,239.8)	1503.2 (1332.0, 1687.6)	0.0 (0.0, 0.0)	671,463.5 (594,430.0, 753,680.8)	848.7 (750.1, 959.3)	0.0 (0.0, 0.0)	40,082.0 (26,620.3, 56,452.9)	50.9 (33.9, 72.2)	−0.1 (−5.7, 6.0)
United Arab Emirates	128,995.8 (111,865.7, 150,111.0)	1540.4 (1357.0, 1746.4)	−0.2 (−0.5, 0.1)	73,631.2 (63,867.3, 85,551.4)	872.6 (766.0, 999.5)	−0.2 (−0.5, 0.1)	4399.3 (2922.0, 6201.4)	52.5 (35.0, 75.3)	−0.1 (−5.2, 5.3)
Yemen	591,183.5 (510,207.9, 683,765.7)	1605.5 (1418.5, 1808.8)	0.1 (0.0, 0.2)	334,423.7 (288,809.9, 389,271.2)	909.9 (799.1, 1038.5)	0.1 (0.0, 0.2)	20,035.9 (13,160.4, 28,849.8)	54.0 (35.6, 76.5)	0.3 (−4.6, 5.6)

Abbreviations: DALY, disability‐adjusted life year; Pcs, percent changes; UI, uncertainty interval.

### 3.2 ASIR and Percentage Changes

The ASIR also varied slightly by country and sex within the region (Figure 1A). Among the countries, Egypt had the highest ASIR for urticaria with ASIR of 1892.4 per 100,000 population (95% UI: 1642.7–2130.7). In contrast, the incidence rate for urticaria in Turkey was 1503.2 per 100,000 population (95% UI: 1332.0–1687.6), as it had the lowest ASIR in 2021. A consistent sex‐based pattern showed that females had higher ASIR rates than males. Among countries with the lowest ASIR, Turkey exhibited significant differences between sexes, with higher rates in women (Table 1).

Figure 1Age‐standardized incidence (A), prevalence (B) and DALYs (C) for Urticaria (per 100,000 population) in the Middle East and North Africa region in 2021, by sex and country. DALY, disability‐adjusted‐life‐years.

**Figure:**
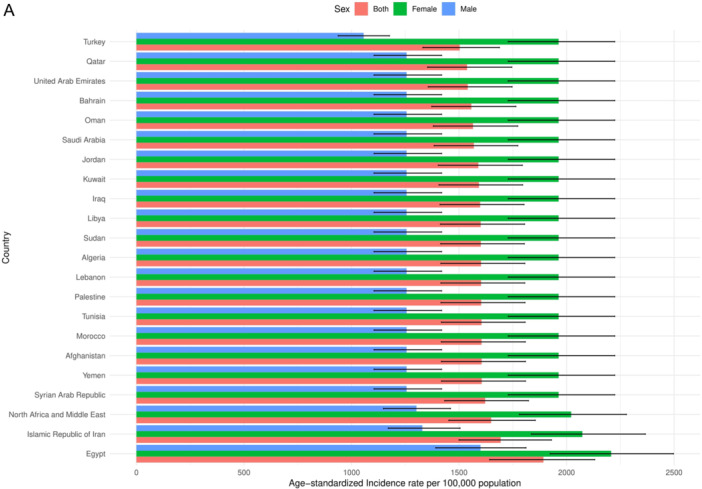

**Figure:**
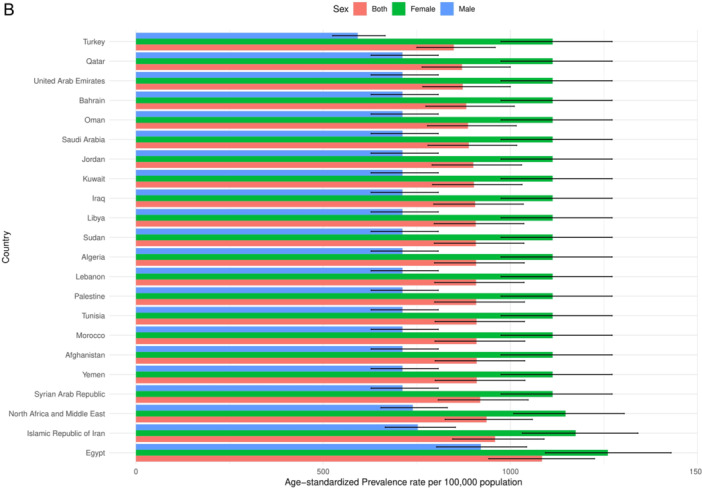

**Figure:**
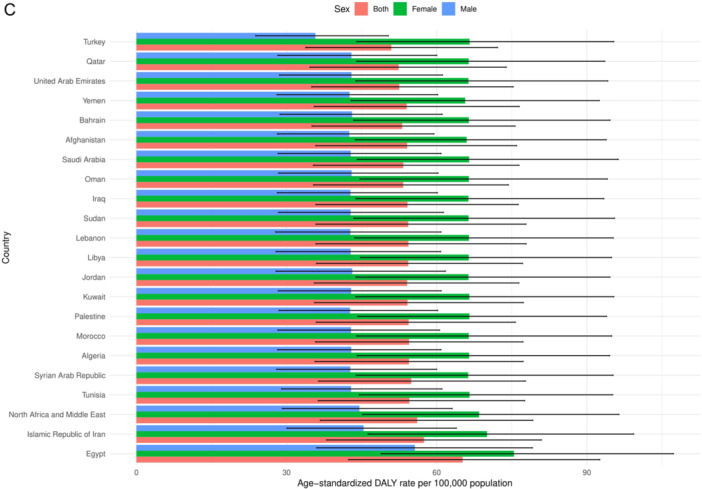

Sex‐stratified data showed that the percentage change in ASIR for both men and women remained consistently similar and close to zero across all countries. In Libya, there was a slight, non‐significant increase in males and a minimal decrease in females; overall, the percentage change in both sexes was similar to other countries. Between 1990 and 2021, the ASIR percentage change slightly decreased in Bahrain, Afghanistan, and Palestine, while it increased in the Syrian Arab Republic and Kuwait (Figure S1).

### 3.3 Age‐Standardized Prevalence Rates (ASPRS) and Percentage Changes

In 2021, Egypt had the highest ASPR in MENA, with an ASPR of 1084.3 per 100,000 population (95% UI: 942.1–1224.4), followed by Iran at 959.0 per 100,000 population (95% UI: 845.8–1089.8) and the Syrian Arab Republic at 919.2 per 100,000 population (95% UI: 807.3–1047.3). In contrast, countries with the lowest ASPRs were Turkey, 848.7 per 100,000 population (95% UI: 750.1–959.3); Qatar, 870.7 per 100,000 population (95% UI: 764.2–999.4); and the United Arab Emirates, 872.6 per 100,000 population (95% UI: 766.0–999.5). A consistent sex‐based pattern revealed that females generally had higher ASPR rates compared to males (Table 1 and Figure 1B).

There were minimal changes in ASPR per 100,000 population from 1990 to 2021, with both men and women showing similar, near‐zero trends across all countries. In Libya, males had a slight increase and females a small decrease, but both trends were comparable to other countries. Bahrain, Afghanistan, and Palestine saw a small decrease in ASIR, while the Syrian Arab Republic and Kuwait showed an increase (Figure S2).

### 3.4 Age‐Standardized DALY Rates and Percentage Changes

There has not been much difference in countries and sexes in the MENA region in terms of age‐standardized DALY rates (Figure 1C). However, Egypt had the highest DALY rate at 65.2 compared to others (95% UI: 42.4–92.6), followed by Iran at 57.5 (95% UI: 38.0–80.9) and Morocco at 54.5 (95% UI: 38.2–77.2), while the lowest rates were seen in Turkey at 50.9 (95% UI: 33.9–72.2), Qatar at 52.4 (95% UI: 34.7–73.9), and Bahrain at 53.1 (95% UI: 35.1–75.6). In most countries, females consistently showed higher DALY rates compared to males (Table 1).

Between 1990 and 2021, percentage changes in the DALY rates remained relatively stable in the MENA countries (Figure S3). The Syrian Arab Republic had the highest increases in DALY rates (0.9%; 95% UI: −4.1 to 6.2), followed by Kuwait (0.8%; 95% UI: −4.5 to 7.0), and Oman (0.3%; 95% UI: −5.3 to 6.0), whereas Afghanistan (−0.8%; 95% UI: −0.6 to 4.7) and Palestine (−0.6%; 95% UI: −6.2 to 5.2) had decreases, which were all non‐significant. These data also indicated a sex disparity, with most countries showing more pronounced percentage decreases in the rates of DALYs for males compared with females (Table 1 and Figure S3).

### 3.5 Age and Sex Patterns

In 2021, the incident cases and rates were highest in the under‐5 age group for both sexes. The number of cases and rates decreased sharply up to the 10–15‐year age group, remained stable until ages 70–75, and then slightly declined. Women had higher incidence cases than men across most age groups up to 60–65, particularly between 10 and 19 years. However, after age 65 through 95+, men exhibited slightly higher incidence cases. Overall, incidence rates were consistently higher in women than in men across all ages (Figure 2A).

Figure 2Total incident cases and Incidence per 100,000 population (A), total prevalent cases and prevalence per 100,000 population (B) and total DALYs and DALY per 100,000 population (C) of urticaria in the Middle East and North Africa region, by age and sex in 2021. DALY, disability‐adjusted‐life‐years.

**Figure:**
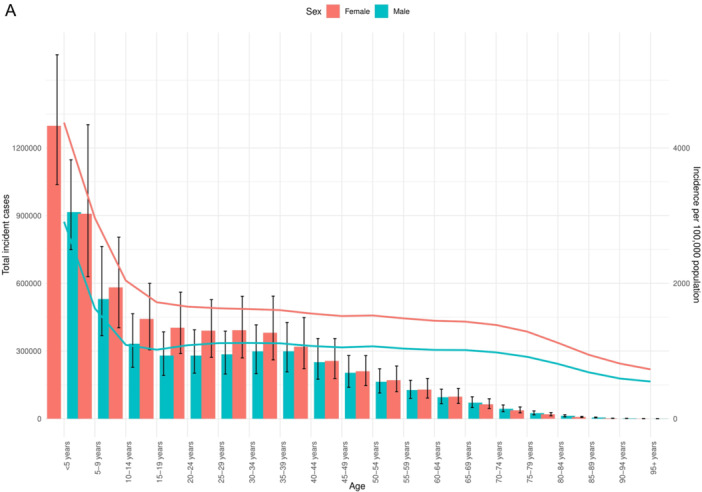

**Figure:**
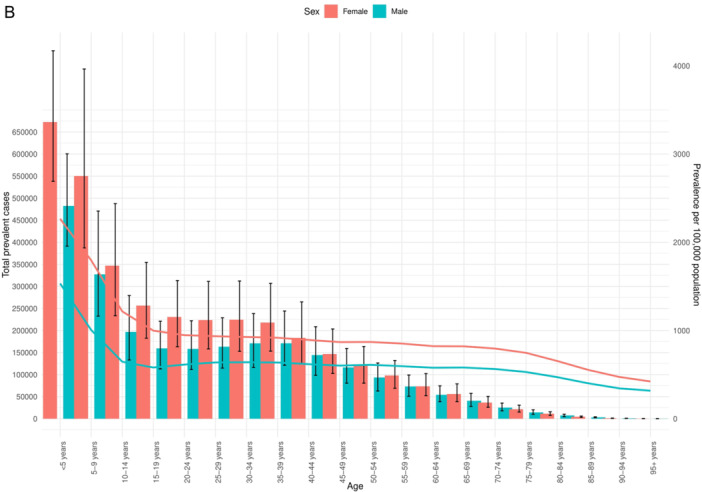

**Figure:**
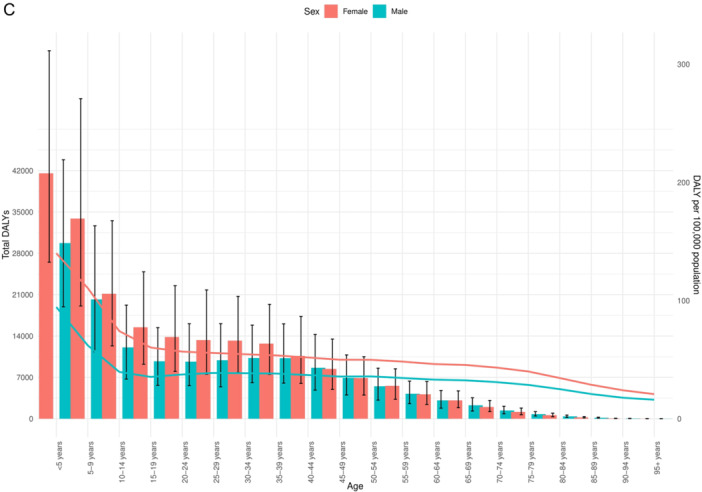

Similarly, the numbers of prevalent cases and rates decreased up to the 10–15‐year age group, remaining stable until ages 70–75, and then declining in older age groups (Figure 2B).

The numbers and rate of regional DALY also decreased with age in both sexes, with the peak in the age group under 5 years for both males and females (Figure 2C). For DALY rates, female experienced higher values than men in all ages. The regional DALYs for age groups after 55 years, males consistently had higher DALYs than females. In age groups less than 55 years old, females had higher DALYs than males.

### 3.6 Association With Socio‐Demographic Index (SDI)

**Figure 3:**
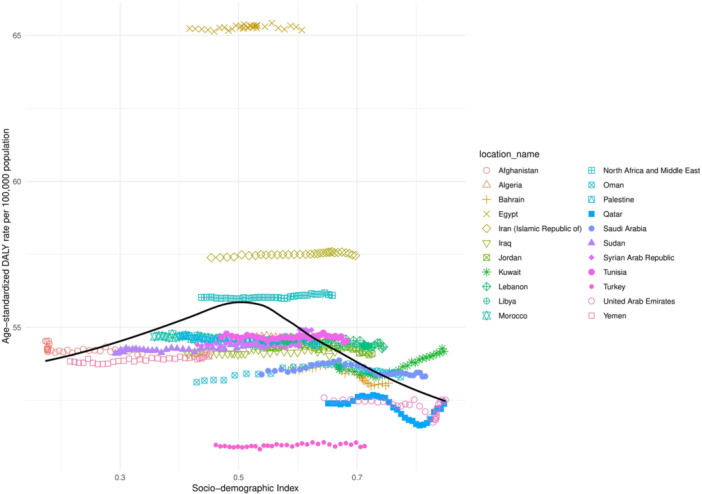
Age−standardized DALY rate per 100,000 population of urticaria for the 21 countries, by SDI; The black line represents expected DALY rates based on the global relationship between SDI and disease burden. Each point on the plot indicates the observed age‐standardized DALY rate for a specific country in 2021. DALY, disability‐adjusted‐life‐years. SDI, socio‐demographic index.

As shown in Figure 3, the black solid line showed that the age‐standardized DALY rates increased with SDI up to 0.5 and then decreased from 0.5 to 1. Countries like Turkey had a lower burden than what would be expected from their SDI, while the reverse was true for Iran and Egypt. Most of the other countries in the MENA region, including Afghanistan, Sudan, Lebanon, and Tunisia, had burdens that were consistent with expectations.

## 4 Discussion

This study aimed to report the burden of urticaria in the MENA region using the most recent GBD 2021 data. In 2021, urticaria accounted for over 10.3 million incident cases, 5.8 million prevalent cases, and more than 353,000 DALYs in the region. The age‐standardized incidence, prevalence, and DALYs rates in MENA did not change substantially between 1990 and 2021. The countries with higher incidence rates also exhibited higher prevalence and DALYs rates. Across the region, Egypt and Iran accounted for the highest burden, while Turkey reported the lowest. Females in total exhibited higher incidence, prevalence, and DALY rates compared to males. The burden of urticaria had a negative association with age; with the highest total incidence, prevalence and DALYs observed in people under 5 years old. Countries with the highest and lowest SDI had lower age‐standardized DALY rates, while countries with mid‐level SDI had the highest peaks.

We found that the burden of urticaria in the MENA region from 1990 to 2021 has remained stable. Pu et al. reported that the age‐standardized incidence, prevalence, and DALY rates of urticaria in 2019 in the MENA region were lower than the global burden of urticaria. They also reported that the burden of urticaria from 1990 to 2019 did not change significantly which aligns with our findings [8]. This could be caused by several factors including differences in environmental exposure in different parts of the world, genetic predispositions, healthcare infrastructure, and reporting methods [22, 23].

Among the MENA countries, Egypt had the highest burden of disease followed by Iran, while Turkey had the lowest. The high rates of urticaria in Egypt and Iran can be explained by increased exposure to dust and pollutants due to the desert climate [23], as well as limited healthcare resources due to economic restraints [24, 25]. Given that chronic urticaria is primarily immune‐mediated, air pollution is unlikely to be a direct cause of the disease. However, emerging evidence suggests that certain pollutants may be associated with worse disease control or increased healthcare utilization in some settings. A recent prospective cohort study reported pollutant‐specific associations, particularly between higher ozone levels and poorer disease control in chronic urticaria patients, rather than a generalized effect of overall pollution burden [26]. Similarly, a time‐series analysis in China found modest correlations between short‐term increases in ozone, PM2.5, and PM10 and outpatient visits for chronic urticaria, although these associations do not establish causality and the effect sizes were small [27]. Taken together, these findings suggest that air pollution might act as a modifier of symptom activity in susceptible individuals rather than a primary trigger of CSU. Higher levels of psychological stress and anxiety may also have a role to play [28]. On the other hand, Turkey's low urticaria rates might be due to its temperate climate, robust healthcare system, healthier Mediterranean diet, lower psychological stress, effective disease management, and public health awareness [29, 30, 31].

Our findings showed that urticaria affected younger populations more than the older ones. This is in line with other studies that suggest children have higher incidence rates among other age groups [2, 6]. This can be due to factors such as an immature immune system, increased sensitivity to allergens, and the development of food or environmental allergies [32, 33]. Viral infections and medication sensitivities also trigger urticaria in these age groups [34]. Genetic predisposition, particularly in families with a history of allergic diseases, plays a role [35]. Young children's sensitive skin and higher exposure to environmental irritants further heighten the risk [36]. Misdiagnosis or delayed recognition may lead to prolonged symptoms and higher DALY rates, as the condition often goes untreated or poorly managed [37].

We also found that women had a higher burden of the disease than men across all age groups. This is in accordance with past research [38, 39]. The high urticaria rates in women might be due to sex hormone fluctuations, greater allergic sensitivity, and higher stress levels [40]. Various cosmetic components, such as fragrances, preservatives, and hair dyes can cause immediate skin reactions, including urticaria. Given that women typically use more cosmetic products, their exposure to these potential triggers is higher [41].

Interestingly, we found that age‐standardized DALY rates had an inverse V‐shape association with SDI, as middle SDI had the highest burden. However, Chu et al. indicated that European countries with poor conditions had higher DALYs for urticaria [42]. These trends could be explained by factors related to SDI and urticaria pathophysiology. In countries with lower SDI, the DALY rates of urticaria tends to be lower despite its weaker health infrastructures as exposure to air pollution and industrial chemicals is lower [43, 44]. As countries reach an intermediate SDI, the effects of urbanization and industrialization become more prominent, increasing exposure to allergens and polluted air, while healthcare systems still have not improved substantially, leading to a higher disease burden [45]. In contrast, high‐SDI countries benefit from well‐developed healthcare systems, better public awareness, and access to effective treatments, which can be associated with a lower burden of urticaria.

This study provides estimates using the latest GBD database in the MENA region. These findings can help policymakers in MENA to better navigate their health expenditure and impose more efficient strategies to reduce the overall burden of diseases in their countries. This study has also some limitations. Data sparsity and underreporting of the MENA region is a major concern which can lead to misdiagnosis and underestimation. Also, environmental and genetic factors unique to the region are often not fully considered. Furthermore, GBD does not differentiate between various types of urticaria (e.g., chronic spontaneous urticaria, inducible urticaria such as solar, cold, aquagenic, and contact urticaria). This generalization prevents an accurate understanding of how different triggers and environmental exposures contribute to disease burden in the region. Lastly, variations in healthcare systems result in different definitions and classifications of urticaria, causing inconsistencies in case reporting.

## 5 Conclusions

Though it differs across countries, overall, the burden of urticaria has not changed significantly since 1990 in the MENA region. Data show that females have higher burden of disease estimates than males especially in younger ages. Countries with mid‐level SDI had the highest rates. This study suggests that policy makers should focus on standardizing reporting and broadening research to strengthen future burden estimates, raising awareness and improving access to healthcare resources and lastly reducing exposure to environmental factors such as pollutants and allergens. Future studies are needed to explore subtype‐specific burdens and provide better estimates for future policies.

## Author Contributions

**Amir Sajad Bagheryan:** conceptualization, investigation, formal analysis, writing – original draft, writing – review and editing, methodology, project administration. **Soroush Oraee:** formal analysis, writing – original draft, writing – review and editing. **Mehra Fekri:** formal analysis, writing – original draft, writing – review and editing. **Farzin Tahmasbi Arashlow:** writing – original draft, writing – review and editing. **Hojjat Layegh:** writing – original draft, writing – review and editing. **Seyed Aria Nejad Ghaderi:** supervision, conceptualization, investigation, formal analysis, writing – original draft, writing – review and editing, methodology, visualization. **Shirin Zaresharifi:** supervision, conceptualization, investigation, formal analysis, writing – original draft, writing – review and editing, methodology. All authors have read and approved the final version of the manuscript. Shirin Zaresharifi had full access to all the data in this study and takes complete responsibility for the integrity of the data and the accuracy of the data analysis.

## Funding

The authors have nothing to report.

## Ethics Statement

Ethics committee of Shahid Beheshti University of Medical Sciences, Tehran, Iran approved the study (ethics code: IR.SBMU.MSP.REC.1403.704).

## Consent

The authors have nothing to report.

## Conflicts of Interest

The authors declare no conflicts of interest.

## Transparency Statement

Shirin Zaresharifi affirms that this manuscript is an honest, accurate, and transparent account of the study being reported; that no important aspects of the study have been omitted; and that any discrepancies from the study as planned have been explained.

## Supporting information


**Figure S1:** The percentage change in age−standardized incidence per 100,000 population of urticaria between 1990 and 2021 in the Middle East and North Africa region, by sex and country.
**Figure S2:** The percentage change in age−standardized prevalence per 100,000 population between 1990 and 2021 of urticaria in the Middle East and North Africa, by sex and country.
**Figure S3:** The percentage change in age−standardized DALY per 100,000 population between 1990 and 2021 of urticaria in the Middle East and North Africa region, by sex and country. DALY = disability‐adjusted‐life‐years.

## Data Availability

The data used for these analyses are all publicly available at https://vizhub.healthdata.org/gbd-results/.
