# Supplementary material for: The Burden of Urticaria in the Middle East and North Africa Region From 1990 to 2021: A Systematic Analysis Based on the GBD Study 2021
Source: Health Sci Rep. 2026 Aug 4;9(8):e72976. doi: 10.1002/hsr2.72976 (PMC13438896; doi:10.1002/hsr2.72976)
Supplement: Supplementary file 1 [file HSR2-9-e72976-s001.docx]

The burden of urticaria in the Middle East and North Africa region from 1990 to 2021: Results from the GBD Study 2021

**Figure S1.** The percentage change in age−standardized incidence per 100,000 population of urticaria between 1990 and 2021 in the Middle East and North Africa region, by sex and country.

**Figure S2.** The percentage change in age−standardized prevalence per 100,000 population between 1990 and 2021 of urticaria in the Middle East and North Africa, by sex and country**.**

**Figure S3.** The percentage change in age−standardized DALY per 100,000 population between 1990 and 2021 of urticaria in the Middle East and North Africa region, by sex and country. DALY = disability-adjusted-life-years.
